# Comprehensive Identification and Expression Analysis of the *SWEET* Gene Family in *Actinidia eriantha* Reveals That Two *AeSWEET11* Genes Function in Sucrose and Hexose Transport

**DOI:** 10.3390/plants14203140

**Published:** 2025-10-11

**Authors:** Xin Feng, Qingqing Huang, Minxia Gao, Ruilian Lai, Yiting Chen

**Affiliations:** 1 Fruit Research Institute, Fujian Academy of Agricultural Sciences, Fuzhou 350013, China; fengxin1506@163.com (X.F.); 18779987006@163.com (Q.H.); gaominxia1206@163.com (M.G.); lairl0618@163.com (R.L.); 2 Research Centre for Engineering Technology of Fujian Deciduous Fruits, Fujian Academy of Agricultural Sciences, Fuzhou 350013, China

**Keywords:** *Actinidia eriantha*, *SWEET* gene family, fruit development and ripening, sugar content, sugar transport activity

## Abstract

Sugars are key metabolites influencing the flavor and quality of kiwifruit, with their accumulation in fruit relying on sugar transporters. Recently identified sugar transporters known as SWEETs play significant roles in modulating plant growth, development, and fruit ripening. However, the characteristics of *SWEET* genes in *Actinidia eriantha* remain poorly understood. In this study, a total of 26 *AeSWEET* genes were identified across 17 chromosomes. These genes encoded proteins ranging from 198 to 305 amino acids in length and contained 5 to 7 transmembrane helices. Both interspecific and intraspecific phylogenetic trees categorized AeSWEET proteins into four distinct clades. The motif and domain structures were conserved within each clade, although variations were observed in exon-intron organizations. One tandem and fourteen segmental duplication events were identified as primary drivers of the *AeSWEET* family expansion. Comparative syntenic mapping showed a closer homology of the *AeSWEET* family with that of dicotyledons compared to monocotyledons. Promoter *cis*-element analysis indicated the potential responses of *AeSWEET* genes to five phytohormones and seven environmental stressors. Quantitative real-time PCR analysis revealed tissue-specific expression profiles of *AeSWEET* genes, with two *AeSWEET11* genes (*AeSWEET11a* and *AeSWEET11b*) showing significantly higher expression levels in fruit tissues. Their expressions were positively correlated with sucrose, fructose, and glucose contents throughout fruit development and ripening. Transient transformation tests in tobacco leaves verified the predominant localization of AeSWEET11a and AeSWEET11b to the plasma membrane. Functional assays in yeast mutants revealed that AeSWEET11a and AeSWEET11b both possessed sucrose and hexose transport activities. These findings highlight the potential of targeting *AeSWEET11a* and *AeSWEET11b* to enhance sugar accumulation in the fruit of *A. eriantha*, thereby providing a foundation for improving the flavor profile of commercial cultivars.

## 1. Introduction

Sugars serve as substrates for various primary metabolites and secondary metabolites, playing critical roles in numerous physiological processes in plants, including energy metabolism, signal transduction, growth and development, and stress responses [1]. Sugars are mainly produced in source tissues like mature leaves, from where they are subsequently transported to sink tissues, with fruits being a primary recipient [2]. Their accumulation in fruit crops not only provides energy for growth but also influences fruit quality and yield [3]. The process involves both sugar metabolism and transport, with sugar transporters serving as key components of the latter [4]. A variety of sugar transporters have been identified, primarily classified into monosaccharide transporters (MSTs), sucrose transporters (SUTs), and Sugars Will Eventually Be Exported Transporters (SWEETs) [5]. MSTs and SUTs share similar topological features and facilitate sugar transport using the electrochemical proton gradient, either in the same or opposite direction as sugar movement [6,7]. In contrast, SWEETs typically function as bidirectional transmembrane transporters, allowing sugars to traverse biological membranes along a pH-independent concentration gradient [8]. SWEETs play a pivotal role in mediating phloem loading in source tissues and unloading in sink tissues, particularly in fruits.

SWEET proteins are ubiquitously found in prokaryotes, animals, and plants, with their biological functions in plants exhibiting greater complexity compared to those in prokaryotes and animals [9]. Plant SWEETs possess the ability to transport various sugars, including sucrose, glucose, fructose, and galactose, thereby contributing to a broad spectrum of biological processes, such as vegetative and reproductive growth, hormone signaling, pathogenesis, abiotic stress responses, and senescence [10,11,12,13,14]. SWEET proteins belong to the MtN3/saliva family (PF03083), characterized by an N-terminus located outside the cytoplasm and a C-terminus positioned inside [15]. Typically, SWEET proteins contain seven transmembrane helices, with the fourth helix being less conserved and primarily serving as a linker that subdivides the protein into two MtN3/saliva domains [9]. Phylogenetic relationships classify plant SWEETs into four distinct clades. Members within the same clade may perform different physiological functions and have distinct organelle localization, yet they typically transport comparable substrates [16]. In general, SWEETs from clades I and II mainly facilitate the transport of hexose sugars, while those in clade III preferentially transport sucrose, and clade IV members favor fructose transport [17].

Unlike the SWEET proteins found in mammals, chordates and bacteria, which are encoded by a single gene, plant SWEETs are encoded by multiple genes [18,19]. Since the characterization of the *AtSWEET* genes in *Arabidopsis* [20], the *SWEET* gene family has been extensively investigated in economically important plants, such as rice [18], peanut [21], pear [22], banana [23], plum [24], and grape [25]. Recently, *SWEET* genes have been demonstrated to play diverse functions during fruit development and ripening. For instance, in melon, *CmSWEET10* has been identified as a sucrose transporter localized on the plasma membrane of phloem sieve elements and companion cells [26]. It exhibits high expression levels during the early stages of sweet melon fruit development, suggesting its involvement in phloem apoplastic unloading and sucrose accumulation [26]. In cucumber, *CsSWEET7a* facilitates sugar unloading from phloem by transferring hexoses from companion cells into the apoplasmic space, thereby enhancing the metabolism of raffinose family oligosaccharides and promoting fruit growth through additional sugar unloading [27]. In tomato, the *SlSWEET15* gene exhibits high expression in developing fruits, with its protein accumulating in vascular tissues and seed coats, key locations for sucrose unloading in fruits, where it mediates the efflux of sucrose from phloem into apoplasm, facilitating subsequent importation into storage parenchyma cells during fruit maturation [28]. In apple, *MdSWEET23* is localized to the plasma membrane, and it is primarily expressed in the vascular bundles of the sepal and carpel [29]. Knock-down of *MdSWEET23* resulted in reduced concentrations of sucrose and sorbitol in the fruit [29].

Kiwifruit (*Actinidia* Lindl.) is a genus of perennial deciduous fruit crops that originated in China and has since achieved global distribution [30]. The fruit is abundant in vitamins, minerals, dietary fiber and other metabolites beneficial to human health, contributing to its high consumer and establishing it as an important economic crop worldwide [31]. Although the genus *Actinidia* comprises 75 taxa, including 56 species and 21 varieties, commercial cultivars primarily derive from *A. chinensis* var. *chinensis* and *A. chinensis* var. *deliciosa*, with limited contributions from *A. arguta* and *A. eriantha* [32]. Notably, *A. eriantha* is characterized by its ease of peeling, convenience for consumption, and higher vitamin C content, which have generated increased interest [33]. However, the low sugar content in the fruit of most *A. eriantha* leads to suboptimal flavor, thereby limiting its market potential. Therefore, investigating sugar metabolism and transport in *A. eriantha* fruit is crucial. *SWEET* genes are known to affect fruit sugar content by modulating sugar transport processes [26,27,34]. Although several *SWEET* genes have been identified in *A. chinensis* [35,36], the characterization and functional roles of *SWEET* family genes in kiwifruits, particularly in *A. eriantha*, remain largely unexplored. In this study, we performed a detailed analysis of the conserved protein domains, evolutionary relationships, gene structures, collinearity patterns, and promoter *cis*-acting elements of the *AeSWEET* gene family in *A. eriantha*. Moreover, a cultivar exhibiting high soluble sugar content in its fruits was employed to investigate the expression patterns of *AeSWEET* genes across different tissues and fruit developmental stages. *AeSWEET11a* and *AeSWEET11b* were identified after correlation analysis between expression levels and sugar contents throughout fruit development and ripening. Their sugar transport capabilities were then demonstrated through functional complementation assays in yeast mutants. Our findings revealed the association of two *AeSWEET* genes with fruit ripening, thereby providing promising candidate genes for molecular breeding programs aimed at enhancing fruit sugar accumulation in *A. eriantha*.

## 2. Results

### 2.1. Identification of the AeSWEET Family Genes in A. eriantha

Through a systematic genome-wide screening approach employing HMMER and BLASTP algorithms, 26 *AeSWEET* genes were identified in *A. eriantha*. These genes were then designated as *AeSWEET1a* to *AeSWEET17c* based on their evolutionary proximity to previously identified *SWEET* genes in related species (Table S1). Subsequent analysis revealed that all 26 AeSWEET proteins contained transmembrane domains, with transmembrane helices (TMHs) ranging from 5 to 7, as presented in Table 1. These AeSWEETs exhibited a wide range of amino acid lengths and molecular weights, spanning from 198 amino acids (AeSWEET4a) to 305 amino acids (AeSWEET6a), and from 21,746.05 Da (AeSWEET4a) to 34,066.5 Da (AeSWEET6a), respectively. The majority of AeSWEETs displayed theoretical isoelectric points (pI) above 7, with the exception observed in AeSWEET17a (pI 6.5), indicating predominantly alkaline characteristics. The instability index of 20 AeSWEET proteins was below 40, indicative of protein stability, while the remaining 6 AeSWEET proteins were deemed unstable. All AeSWEET proteins exhibited grand average of hydropathy (GRAVY) values above 0.24, suggesting their hydrophobic nature. Additionally, subcellular localization predictions indicated that all AeSWEETs were localized to the plasma membrane.

### 2.2. Chromosomal Mapping and Evolutionary Analysis of AeSWEETs

**Figure 1:**
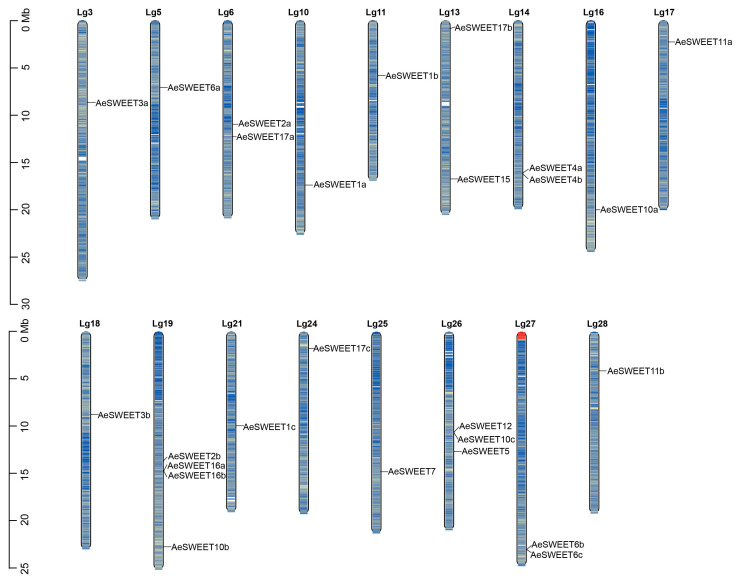
Distribution of *AeSWEETs* on the whole-genome chromosomes. The scale represented chromosome length, with the black lines denoting the loci of individual *AeSWEET* genes. Chromosome coloration reflected gene density, where blue denoted high gene density and yellow signified low gene density.

The chromosomal localization of *AeSWEET* genes was determined using the *A*. *eriantha* genome published in 2023 [37]. The results revealed an uneven distribution of these genes across 17 of the 29 chromosomes (Lgs) with gene counts ranging from one to four. As shown in Figure 1, the highest concentration was found on chromosome Lg19, which contained four genes, followed by Lg26 with three genes, and four chromosomes (Lg6, Lg13, Lg14, and Lg27) each containing two genes. The remaining 11 chromosomes each contained a single gene. Notably, both *AeSWEET6b* and *6c* were located on Lg27, exhibiting identical transcriptional orientations. Their intergenic spacer region spanned just 500 bp and harbored no other functional genes (Table S1). Given their high sequence similarity (73.33%; Table S2), *AeSWEET6b* and *6c* likely arose from tandem duplication. Additionally, gene density analysis showed that the *AeSWEET* genes were mainly located in genomic regions with relatively high gene density.

**Figure 2:**
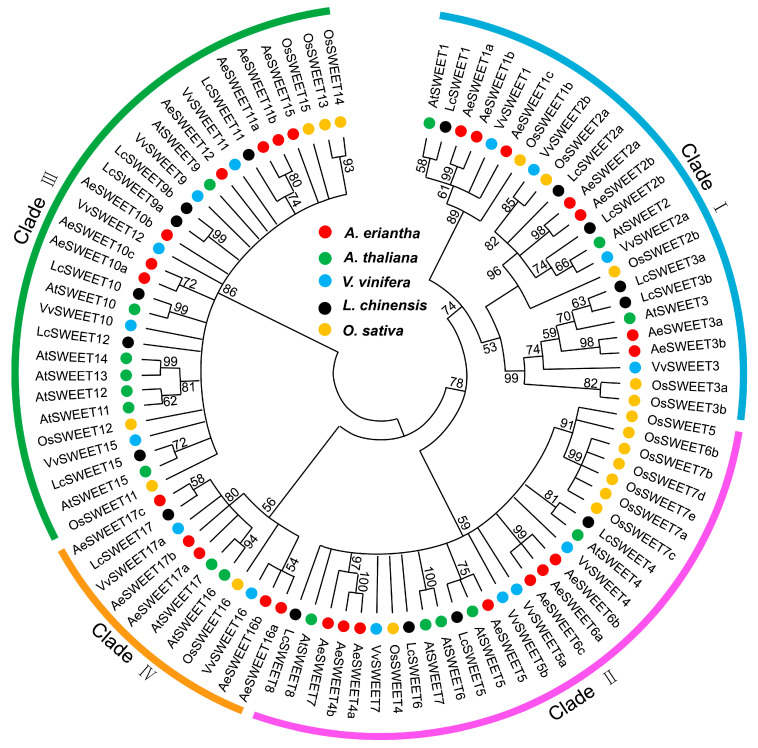
Phylogenetic relationships of AeSWEETs and other plant SWEETs. SWEET members from *Actinidia eriantha* (Ae), *Arabidopsis thaliana* (At), *Vitis vinifera* (Vv), *Litchi chinensis* (Lc), and *Oryza sativa* (Os) were marked in the tree with red, green, blue, black and orange dots, respectively. The maximum likelihood phylogenetic tree was constructed using the LG model. Node numbers show the bootstrap values from 1000 replicates.

Subsequently, an interspecific phylogenetic tree was constructed using the aligned dataset of both dicot and monocot SWEET sequences to assess evolutionary relationships. All SWEET proteins were grouped into four major clades: Clade I, II, III and IV (Figure 2). The AeSWEET proteins were distributed across all clades. Specifically, seven AeSWEETs clustered with plant SWEET1, 2, and 3 in Clade I; another seven were associated with SWEET4, 5, 6, and 7 in Clade II; seven more grouped with SWEET9 to 15 in Clade III; and five aligned with SWEET16 and 17 in Clade IV. Notably, most AeSWEETs within each clade exhibited species-specific clustering, such as AeSWEET1a and 1b in Clade I, AeSWEET6a, 6b and 6c in Clade II, AeSWEET11a and 11b in Clade III, and AeSWEET16a and 16b in Clade IV. Pairwise comparisons of both ORF and protein sequences demonstrated higher sequence identity among *AeSWEET* genes within the same species-specific clade (Table S2). These findings suggested that the expansion of the *AeSWEET* family largely occurred after the divergence of kiwifruit and other plant species.

### 2.3. Protein Motifs, Conserved Domains and Gene Structure Features of AeSWEETs

**Figure 3:**
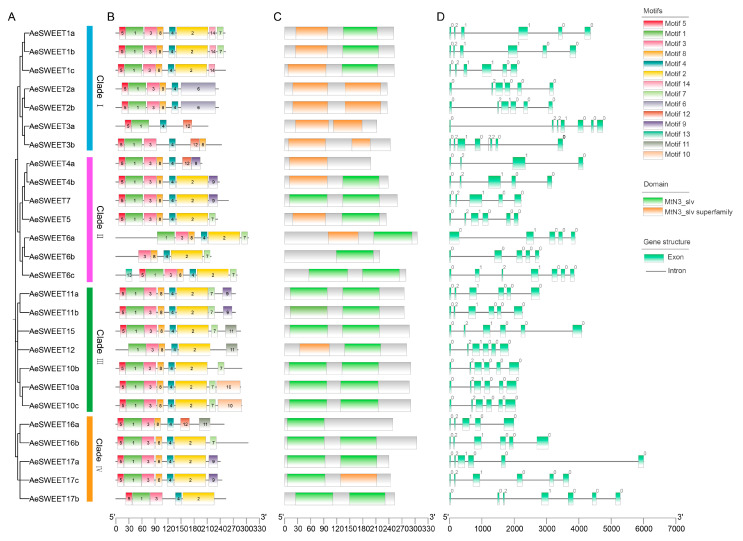
Intraspecific evolutionary tree, conserved motif, functional domain, and gene structure of *AeSWEETs*. (**A**) The phylogenetic tree of 26 *AeSWEETs*. (**B**) The distribution of conserved motifs among *AeSWEETs*. (**C**) The conserved domains of *AeSWEETs*. (**D**) The exon-intron organization of *AeSWEETs*, where a value of 0 indicated that intron insertion did not disrupt the codon, 1 signified intron insertion following the first base of the codon, and 2 denoted intron insertion after the second base of the codon.

Intraspecific phylogenetic analysis grouped the 26 AeSWEETs into four main clades (Figure 3A), consistent with the interspecific phylogenetic tree results. Using the MEME online tool, 14 conserved motifs were identified among the AeSWEET proteins (Figure 3B and Figure S1). Variations in the number and distribution of motifs were observed, with each AeSWEET member containing between 4 and 8 motifs, and unique motif patterns distinguishing the different clades. Specifically, all AeSWEETs in Clade I contained motifs 1, 4 and 5; Clade II members possessed motifs 3, 4, and 8; Clade III included motifs 1, 2, 3, 4, and 8; while Clade IV featured motifs 1, 3, 4, and 5. These distinct motif patterns revealed potentially functional diversity across the AeSWEET clades. Furthermore, conserved domain analysis revealed the presence of MtN3_slv and/or MtN3_slv superfamily domains in AeSWEET proteins (Figure 3C), indicating evolutionary conservation. Additionally, a structural map was generated based on genomic sequences to illustrate the exon-intron arrangements of *AeSWEET* genes (Figure 3D). The number of exons (ranging from 4 to 8) and introns (ranging from 3 to 7) varied significantly among *AeSWEET* family genes, suggesting that intron gain and loss events had occurred during evolution.

### 2.4. Collinearity and Synteny Patterns of AeSWEETs

**Figure 4:**
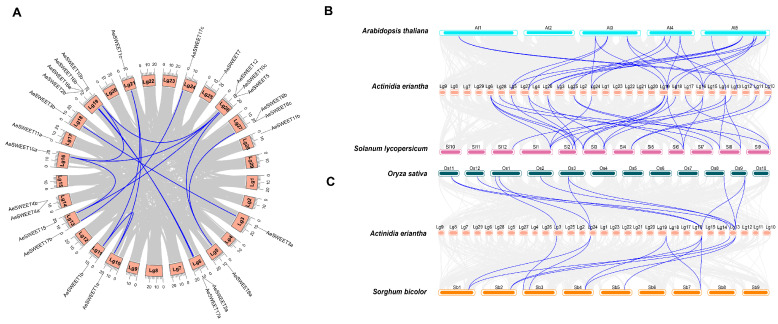
Collinearity analysis of the *AeSWEET* family genes. (**A**) Collinear gene pairs of *AeSWEET* family genes. (**B**) Collinear gene pairs of *SWEETs* across *Actinidia erientha*, *Arabidopsis thaliana*, and *Solanum lycopersicum*. (**C**) Collinear gene pairs of *SWEETs* among *Actinidia erientha*, *Oryza sativa*, and *Sorghum bicolor*. Gray lines were used to connect the collinear gene blocks, while blue lines were utilized to link collinear *SWEET* gene pairs.

An intraspecific collinear analysis was conducted to investigate the potential impact of gene duplication events on the *AeSWEET* family evolution (Figure 4A). Fourteen collinear gene pairs were identified among the *AeSWEETs*, suggesting that segmental duplication events played roles in enhancing genetic diversity. Additionally, certain genes participated in multiple gene pairs, such as *AeSWEET1a*-*AeSWEET1b* and *AeSWEET1a*-*AeSWEET1c*. The nonsynonymous (Ka) and synonymous (Ks) substitution rates were calculated for each duplicated *AeSWEET* gene pair to evaluate selection pressures (Table S3). The Ka/Ks ratios for 13 gene pairs were determined to be less than 1, except for *AeSWEET10a*-*AeSWEET12*, for which the Ka/Ks value could not be calculated due to its high sequence divergence value (pS ≥ 0.75). These findings indicated that the majority of *AeSWEET* genes underwent purifying selection.

Furthermore, the interspecific syntenic maps of *A*. *eriantha* between two representative dicotyledons (*A*. *thaliana* and *Solanum lycopersicum*) and two monocotyledons (*O*. *sativa* and *Sorghum bicolor*) were also performed to identify orthologous genes (Figure 4B,C). The *AeSWEET* genes exhibited significant collinearity with *SWEET* genes from other dicotyledons, displaying the strongest collinear relationship with *S*. *lycopersicum* (28 pairs), followed by *A*. *thaliana* (22 pairs). In contrast, *AeSWEET* genes shared fewer genes with the monocotyledons, with both *O*. *sativa* and *S*. *bicolor* presenting 11 pairs. However, five members (*AeSWEET1c*, *2a*, *10c*, *12*, and *17a*) did not exhibit synteny with any of the four species analyzed, suggesting that they might have emerged after the divergence of these plant lineages.

### 2.5. Diverse Cis-Acting Elements Present in the Promoters of AeSWEET Genes

**Figure 5:**
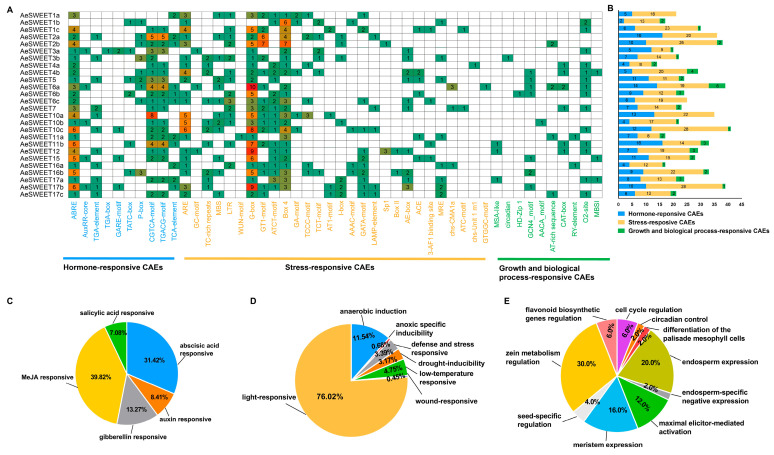
*Cis*-acting elements (CAEs) in the *AeSWEET* promoters. (**A**) Categorization of *cis*-acting elements in *AeSWEET* promoters. Green in the heatmap indicated lower values, while red indicated higher values. (**B**) The counts of *cis*-acting elements within each promoter from the three distinct groups. (**C**) Percentage of *cis*-acting elements associated with hormone-response. (**D**) Percentage of *cis*-acting elements associated with stress-response. (**E**) Percentage of *cis*-acting elements associated with growth and biological processes.

To explore the potential functional characteristics of the *AeSWEET* promoters, a 2000 bp region upstream of the start codon was extracted from each gene and subjected to analysis using PlantCARE. Abundant *cis*-acting elements (CAEs) were identified, comprising ten types of hormone-responsive CAEs, twenty-eight types of stress-responsive CAEs, and ten types of growth and biological process-responsive CAEs (Figure 5A). Hormone-responsive CAEs, such as MeJA, abscisic acid, salicylic acid, auxin, and gibberellin responsive elements, were present in the *AeSWEET* promoters (Figure 5B,C), indicating their broad involvement in hormonal responses. Stress-responsive CAEs were predominantly associated with light response (76.02%), followed by anaerobic induction (11.54%), low-temperature (4.75%), defense and stress (3.39%), drought (3.17%), anoxic specific inducibility (0.68%), and wound (0.45%), highlighting the participation of *AeSWEETs* in abiotic stresses (Figure 5D). Moreover, growth and biological process-responsive CAEs, such as those involved in cell cycle regulation, circadian control, and various metabolic pathways, were found in most *AeSWEET* promoters (Figure 5E). These findings suggest that *AeSWEET* genes play vital roles in diverse biological processes, as well as in responses to phytohormones and environmental stress.

### 2.6. Tissue Expression Patterns of the AeSWEET Family Genes

**Figure 6:**
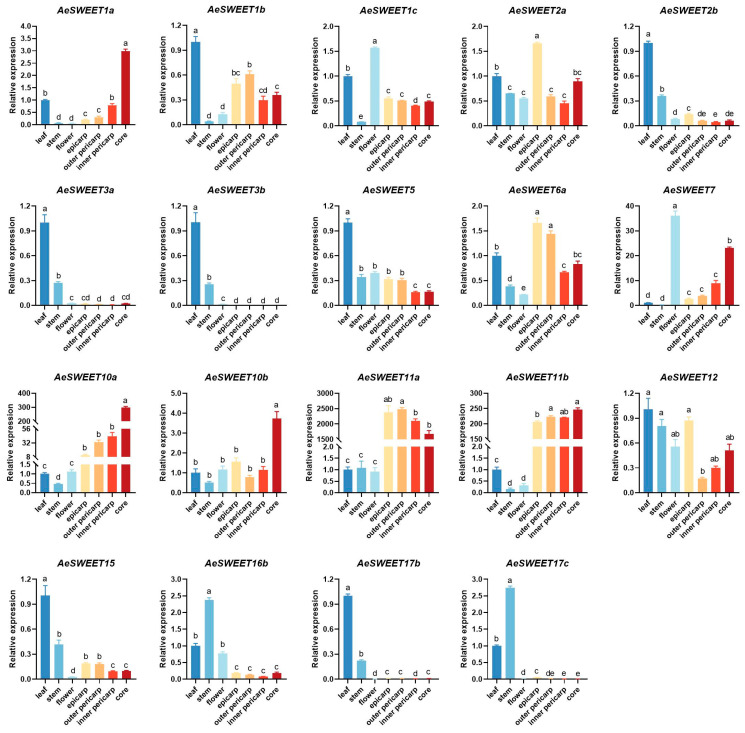
Relative expression of the *AeSWEET* family genes in different tissues. The expression level in the leaf was normalized to 1. Differences in gene expression were assessed by one-way ANOVA with Tukey’s Honestly Significant Difference test, using distinct letters to denote statistical significance (*p* < 0.05).

To investigate the expression patterns of *AeSWEET* family genes comprehensively, qRT-PCR analysis was conducted to detect their expression levels across seven distinct tissues (leaf, stem, flower, epicarp, outer pericarp, inner pericarp, and core). As shown in Figure 6, 19 *AeSWEET* genes were detected to express in at least one tested tissue, while the transcriptional abundance of other seven genes (*AeSWEET4a*, *4b*, *6b*, *6c*, *10c*, *16a*, and *17a*) showed negligible expression (Ct > 35). Eight *AeSWEET* genes, including four (*AeSWEET1b*, *2b*, *3a*, and *3b*) in clade I, one (*AeSWEET5*) in clade II, two (*AeSWEET12* and *15*) in clade III, and one (*AeSWEET17b*) in clade IV, showed higher expression abundance in leaves relative to other tissues, suggesting their involvement in leaf sugar transport or loading processes. Conversely, two genes in clade IV (*AeSWEET16b* and *17c*) displayed peak expression abundance in stems, implying their specialized functions in stem sugar transport. Moreover, *AeSWEET1c* in clade I and *AeSWEET7* in clade II exhibited predominant expression in flowers, suggesting their potential roles in flower sugar transport. Additionally, seven genes, including two in clade I (*AeSWEET1a* and *2a*), one in clade II (*AeSWEET6a*), and four in clade III (*AeSWEET10a*, *10b*, *11a*, and *11b*), manifested elevated expression levels in at least one fruit tissues (epicarp, outer pericarp, inner pericarp, and core). Interestingly, *AeSWEET11a* and *AeSWEET11b* were significantly upregulated in all four fruit tissues, surpassing leaf expression abundance by over 200-fold, suggesting their pivotal involvement in fruit sugar transport mechanism.

### 2.7. Differential Expression of AeSWEET Genes During Fruit Development and Ripening

**Figure 7:**
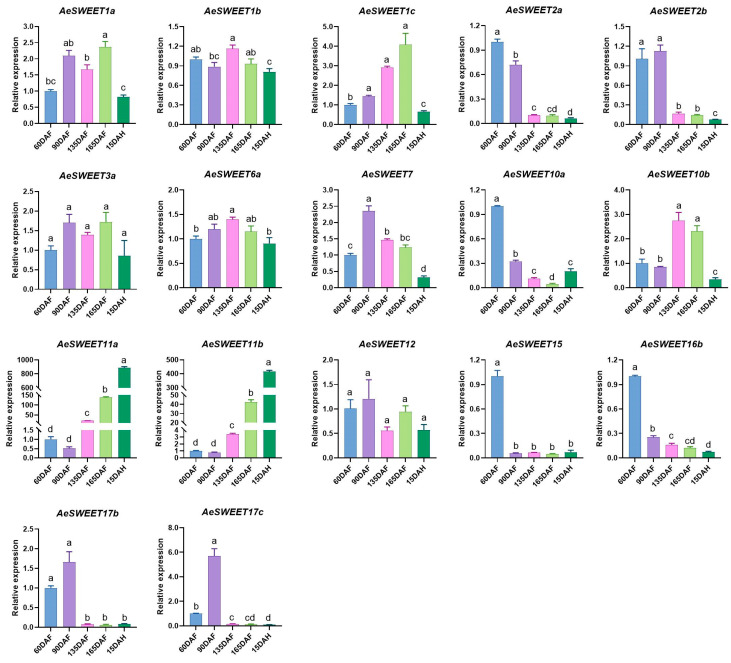
Relative expression of the *AeSWEET* family genes throughout fruit development and ripening. The expression level at 60 days after full bloom was normalized to 1. Differences in gene expression were assessed by one-way ANOVA with Tukey’s Honestly Significant Difference test, using distinct letters to denote statistical significance (*p* < 0.05). DAF indicated days after full bloom, while DAH referred to days after harvest.

To further elucidate the potential roles of *AeSWEET* genes in fruit development and ripening, we evaluated their expression profiles across five distinct developmental stages using qRT-PCR. 15 out of the total 26 *AeSWEET* genes exhibited statistically significant differential expression in at least one of the fruit developmental stages, while *AeSWEET3a* and *AeSWEET12* displayed marginal fluctuations during fruit development and ripening without reaching statistical significance (Figure 7). Eight members (*AeSWEET2a*, *2b*, 7, *10a*, *15*, *16b*, *17b*, and *17c*) showed peak expression levels at 60 DAF or 90 DAF, followed by a decline as the fruit progressed towards maturity. Conversely, five genes (*AeSWEET1a*, *1b*, *1c*, *6a*, and *10b*) exhibited fluctuating increases prior to fruit harvest (165 DAF) and were subsequently downregulated upon fruit ripening (15 DAH). Notably, the expression levels of *AeSWEET11a* and *AeSWEET11b* exhibited significant increments post-90 DAF, peaking at full ripeness (15 DAH), reaching 885-fold and 415-fold higher than those at 60 DAF, respectively. These findings suggest that sugar transport processes during *A*. *eriantha* fruit development and ripening were probably governed by distinct *AeSWEET* gene members. The majority participated in sugar transport pre-harvest, whereas *AeSWEET11a* and *AeSWEET11b* were predominantly engaged in sugar transport throughout fruit development and ripening.

### 2.8. Correlation Analysis of AeSWEET Gene Expression and Sugar Contents During Fruit Development and Ripening

**Figure 8:**
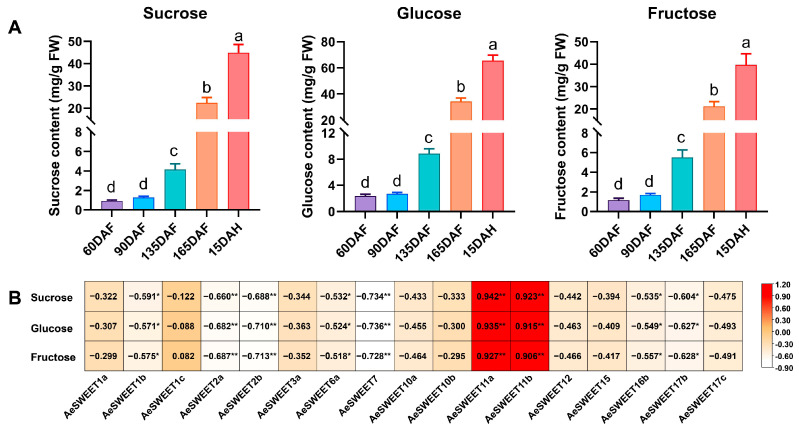
Analysis of sugar contents and their correlation with *AeSWEET* genes during fruit development and ripening. (**A**) The concentrations of sucrose, glucose and fructose during fruit development and ripening. DAF indicated days after full bloom, while DAH referred to days after harvest. Differences in sugar concentrations were assessed by one-way ANOVA with Tukey’s Honestly Significant Difference test, using distinct letters to denote statistical significance (*p* < 0.05). (**B**) Correlation of sugar contents and expression levels of *AeSWEET* genes during fruit development and ripening. Asterisks indicated statistical significance (* *p* < 0.05, ** *p* < 0.01).

The biosynthesis, transport, and metabolism of sugars are critical processes in fruit development and ripening. Sucrose, fructose, and glucose constitute the main soluble sugars found in fruits and are closely associated with kiwifruit quality. The variations in the concentrations of these three sugars at different developmental stages were determined (Figure 8A). At 90 DAF, the levels of the three sugars were slightly elevated compared to 60 DAF, although this increase was not statistically significant. As fruit development and ripening progressed, the concentrations of these sugars significantly increased, peaking at full ripeness (15 DAH). To investigate the relationship between sugar contents and the expression levels of *AeSWEET* genes, Pearson correlation analysis was conducted (Figure 8B). The results showed that the correlation coefficients of 0.927 to 0.942 between *AeSWEET11a* expression levels and the contents of sucrose, glucose, and fructose. Similarly, the *AeSWEET11b* expression exhibited correlation coefficients ranging from 0.906 to 0.923 with the same sugars, indicating that the expression levels of *AeSWEET11a* and *AeSWEET11b* were positively correlated with the contents of these sugars during fruit development and ripening.

### 2.9. Subcellular Localization of AeSWEET11a and AeSWEET11b

**Figure 9:**
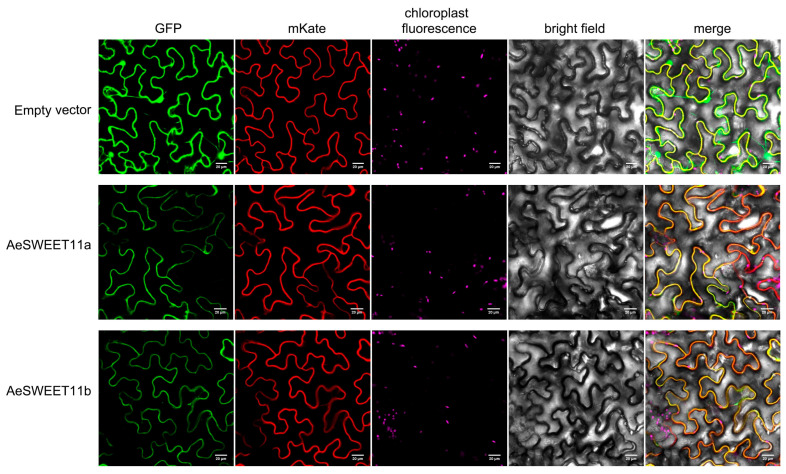
Subcellular localization of AeSWEET11a and AeSWEET11b in *Nicotiana benthamiana* leaves. The vectors 35S:AeSWEET11a-GFP and 35S:AtNAA60-mKate vectors, 35S:AeSWEET11b-GFP and 35S:AtNAA60-mKate vectors, as well as 35S:GFP and 35S:AtNAA60-mKate were independently co-transfected into the tobacco leaves. Scale bars represented 20 μm.

Plant structural genes typically function within distinct subcellular compartments. To validate the presumed subcellular localization, the epidermal cells of *Nicotiana benthamiana* leaves were co-transformed with either 35S:AeSWEET11a-GFP or 35S:AeSWEET11b-GFP and a plasma membrane marker protein labeled with mKate (35S:AtNAA60-mKate) [38]. The control protein (35S:GFP) showed green fluorescence signal distributed throughout the entire cell, including membranes, cytoplasm, and nuclei. In contrast, the green fluorescence signal of 35S:AeSWEET11a-GFP or 35S:AeSWEET11b-GFP was exclusively observed in the membranes, coinciding with the red fluorescence signal of 35S:AtNAA60-mKate (Figure 9).

### 2.10. Functions of AeSWEET11a and AeSWEET11b in Sugar Transporting

**Figure 10:**
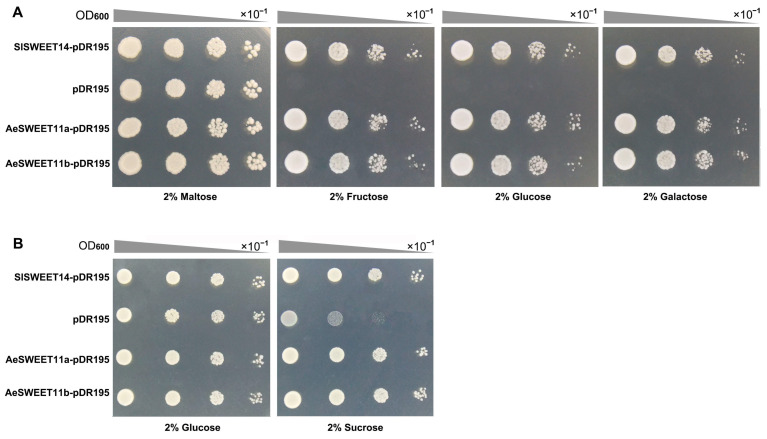
Functional validation of *AeSWEET11a* and *AeSWEET11b* in yeast mutant cells. (**A**) The yeast mutant EBY.VW4000, transformed with AeSWEET11a-pDR195, AeSWEET11b-pDR195, SlSWEET14-pDR195 (positive control), or pDR195 (negative control), was cultured on SD/-Ura solid medium supplemented with 2% (*w*/*v*) maltose, fructose, glucose, or galactose as the sole carbon source. Serial 10-fold dilutions were spotted onto the solid medium. (**B**) The yeast mutant SUSY7/ura3 harboring AeSWEET11a-pDR195, AeSWEET11b-pDR195, SlSWEET14-pDR195 (positive control), or pDR195 (negative control) was grown on SD/-Ura solid medium with 2% (*w*/*v*) glucose or sucrose as the exclusive carbon source.

The functionality of the two *AeSWEET11* genes in sugar transport was tested via heterologous expression in two yeast mutant strains, EBY.VW4000 [39] and SUSY7/ura3 [40]. EBY.VW4000 is a hexose transport-deficient strain incapable of utilizing monosaccharides for growth but can thrive on maltose. In contrast, the SUSY7/ura3 strain lacks extracellular invertase, which prevents it from using sucrose as the sole carbon source, although it possesses sucrose synthase activity and thus can metabolize sucrose via the exogenous sucrose transporter. Spotting assays showed that EBY.VW4000 cells transformed with AeSWEET11a-pDR195, AeSWEET11b-pDR195, or SlSWEET14-pDR195 (the positive control) exhibited robust growth on SD/-Ura medium supplemented with 2% (*w*/*v*) maltose or three monosaccharides (fructose, glucose, and galactose; Figure 10A). Conversely, EBY.VW4000 cells transformed with the empty pDR195 vector (the negative control) only grew on maltose and failed to grow on media containing fructose, glucose, or galactose. Furthermore, SUSY7/ura3 strains transformed with AeSWEET11a-pDR195, AeSWEET11b-pDR195, or SlSWEET14-pDR195 exhibited obviously faster growth on sucrose-containing media than those harboring the empty pDR195 vector (Figure 10B). These results suggested that both *AeSWEET11a* and *AeSWEET11b* functioned as transporters for hexoses (fructose, glucose, and galactose) and sucrose.

## 3. Discussion

### 3.1. The Characteristics of AeSWEET Family Genes in A. eriantha

SWEET proteins play crucial roles in plant growth, development, and defense mechanisms as they aid in the efficient long-distance translocation of sugars from source (such as mature leaves) to sink organs (e.g., seeds and fruits) [9]. These proteins are encoded by a gene family, the size of which varies across species. For instance, litchi contains 16 *LcSWEETs* [41], grape has 17 *VvSWEETs* [42], pear harbors 18 *PbSWEETs* [22], and banana contains 25 *MaSWEETs* [23]. In this study, 26 *AeSWEET* genes were identified in *A*. *eriantha*, which is more than the 17 *AtSWEETs* found in *A*. *thaliana* (a dicotyledonous model plant) [43] and the 21 *OsSWEETs* present in *O*. *sativa* (a monocotyledonous model plant) [18]. Most *AeSWEET* genes encoded basic proteins with 7 transmembrane helices although two proteins contained only 5 to 6 transmembrane helices, similar results were also found in PpSWEET proteins of *Poa pratensis* [44]. There were 4 to 8 exons existing in the *AeSWEET* family, unlike the 5 to 7 exons found in grape *VvSWEET* family [42].

Plant *SWEET* families usually undergo expansion and loss of members throughout evolution. The increased number of *SWEET* genes in higher plants primarily stems from tandem duplication and segmental duplication [45,46]. The proportions of these two duplication mechanisms reveal a striking divergence among species, exhibiting a phenomenon of species-specific dominance [47]. For instance, in *Glycine max*, segmental duplication events gave rise to 69.8% of *GmSWEET* genes, while in *Eucalyptus grandis*, tandem duplication produced 52.0% of *EgSWEET* genes [47]. Similarly, a tandem duplication event within the *A. eriantha* genome specifically produced two *AeSWEET* genes (*AeSWEET6b* and *6c*), while the identification of 14 collinear gene pairs within the *AeSWEET* family strongly suggested that segmental duplication primarily drove the expansion of this family. Additionally, a significantly greater sharing of *SWEET* gene pairs was detected between *A. eriantha* and other dicotyledons compared to monocotyledons. Conversely, Pan et al. [48] observed that the *AsSWEET* family in common oat exhibited stronger collinearity with *SWEET* genes from other monocots than with those of dicots, implying that the divergence of *SWEET* family genes among species occurred synchronously with the split of monocot and dicot lineages. Phylogenetic trees classified the *AeSWEET* family into four distinct clades, corroborating previous studies [9]. However, compared to *A. thaliana*, *A. eriantha* lacked *SWEET8*, *SWEET9*, *SWEET13*, and *SWEET14*. This absence pattern was echoed in other species, such as litchi, which was missing *SWEET7*, *SWEET13*, *SWEET14*, and *SWEET16* [41], and several *Poaceae* crops that lacked *SWEET8* and *SWEET9* [48]. These findings collectively indicated selective loss of specific *SWEET* family members throughout evolution. The loss of members within plant gene families has found to be associated with multiple factors, such as ecological adaptation pressures, functional redundancy, and pseudogenization [49,50]. Furthermore, the Ka/Ks value clearly demonstrated that purifying selection served as the primary evolutionary force shaping *AeSWEET* genes, likely playing a vital role in preserving ancestral biological functions.

### 3.2. Expression and Functional Diversity of AeSWEET Genes

SWEET family genes exhibit differential expression across plant tissues and are integral to the transport of various sugars. The expression patterns of these genes are closely tied to their functional roles, allowing tissue-specific analyses to serve as predictors of biological functions. In rice, the genes *OsSWEET11* and *OsSWEET15* exhibited highly concentrated and specific expressions in caryopses, where they influenced starch accumulation in the pericarp [51]. In *Arabidopsis*, *AtSWEET16* was predominantly expressed in root vacuoles, and mutations in this gene impaired root growth under conditions of excess fructose [43]. Similarly, *NECI*, the *Petunia* homolog of *AtSWEET9*, exhibited predominant expression in nectaries [52]; its silencing led to male sterility and reduced nectar secretion [53,54]. Here, eight *AeSWEET* genes were identified with high expression in leaves, two showed peak expression in stems, another two were predominantly expressed in flowers, and seven exhibited elevated expression in at least one fruit tissue (Figure 6), indicating that sugar transport in different tissues was dominated by distinct *AeSWEET* genes. In addition, 15 *AeSWEET* genes were highly expressed at different time points of fruit development and ripening. However, only two genes exhibited a positive correlation between their expression levels and sugar content, whereas the rest showed negative correlations (Figure 8). The latter suggests a potential role in sugar efflux, which warrants further investigation. Although the widespread expression of *AeSWEET* genes suggest potential metabolic significance, their precise biological roles require further validation through multiple methodologies such as RNA in situ hybridization, immunolocalization, gene overexpression, or VIGS experiments in future investigations.

For the four distinct evolutionary clades characterized, all reported plant SWEET families; however, gene expression patterns within the same clade diverged across species. In *Hemerocallis citrina*, *HcSWEET* genes from identical clades generally shared similar expression profiles [55]. However, in *A. eriantha*, *AeSWEET* genes within the same clade manifested divergent expression modes (Figure 6). This expression divergence proved unsurprising, as *MdSWEET15a* in apple also exhibited high expression in flowers, fruits, and mature leaves, while *MdSWEET12a*, a fellow clade III member, predominantly expressed in fruit tissues [56]. Within *A. thaliana* clade III, *AtSWEET11*, *12*, and *15* mediated sucrose efflux from the seed coat to the embryo [57], whereas *AtSWEET9* specifically transported sucrose out of nectary parenchyma cells [54], indicating functional differentiation among clade members. Furthermore, duplicated genes also tended to diverge in expression [58,59], as evidenced in the *AeSWEET* gene duplicates. For instance, *AeSWEET1a* and *1b* clustered within the species-specific group of clade I, consistent with their features as syntenic gene pairs boasting high sequence similarity (Figure 2, Tables S2 and S3), yet their expression patterns differed from one another (Figure 6). Similarly, duplicated *HvSWEET* genes in barley exhibited differential expression patterns, which was considered to undergo neofunctionalization post-duplication [60]. Additionally, the *AeSWEET* promoters featured *cis*-acting elements responsive to phytohormones, environmental stresses, as well as growth and biological processes (Figure 5), mirroring findings in the promoters of the plum *PsSWEET* family [24] and the peanut *AhSWEET* family [21]. *Cis*-acting elements in plant *SWEET* promoters can interact with transcription factors to modulate gene expression [21,34]. The types and numbers of *cis*-elements varied among distinct *AeSWEET* genes (Figure 5), potentially accounting for their differential expression at the transcriptional level. The *VvSWEET* promoters in grape clade III exhibited a higher abundance of ABRE elements [42], a pattern similarly observed within clade III of the *AeSWEET* family, implying that this subclade likely participated in multiple biological processes via the ABA signaling pathway.

### 3.3. AeSWEET11a and AeSWEET11b Probably Participate in Sugar Transport and Accumulation During Fruit Development and Ripening

Plant *SWEET* genes are crucial for fruit ripening and for regulating sugar accumulating in fruits. In watermelon, *ClSWEET3* emerged as the most abundantly expressed *SWEET* gene throughout fruit development, with its expression levels positively correlating with sugar content [61]. Overexpression of *ClSWEET3* resulted in increased sugar levels, whereas CRISPR-mediated *clsweet3* mutants displayed decreased sugar content and biomass [61]. Although seven *AeSWEET* genes were detected as differentially expressed in at least one fruit tissue (Figure 6), only *AeSWEET11a* and *AeSWEET11b* consistently maintained elevated expression throughout fruit development and ripening (Figure 7), suggesting their crucial involvement in fruit sugar transport. Sucrose, glucose, and fructose constituted the primary soluble sugars defining the characteristic flavor profile of *A. eriantha* fruits [62]. These key sugars progressively accumulated during fruit development, culminating in peak levels upon full ripeness (Figure 8A). And they were significantly and positively correlated with the expression levels of *AeSWEET11a* and *AeSWEET11b* (Figure 8B). *AcSWEET9b* of *A*. *chinensis* var. ‘Donghong’, a homolog of *AeSWEET11b*, was shown to influence sucrose concentration during fruit development via gene overexpression and silencing [35], while its sugar transport activity remains unclear. In apple, *MdSWEET12a* exhibited dominant expression in fruit tissues, correlating positively with sucrose accumulation, and was validated as a sucrose transporter [56]. Generally, *SWEETs* in clades I, II, and IV preferentially transport hexoses, while those in clade III specialize in sucrose transport [63]. Recent studies have demonstrated that individual SWEET protein can simultaneously transport multiple types of sugar. For instance, *Arabidopsis AtSWEET13* can transport both glucose and sucrose [64], while *SlSWEET7a* and *SlSWEET14* in tomato are capable of transporting fructose, glucose, and sucrose [65]. Similarly, *AeSWEET11a* and *AeSWEET11b* in this study were confirmed to transport both hexoses and sucrose (Figure 10). These findings indicate that plant *SWEETs* within clade III have undergone functional differentiation during evolution.

The ORF and protein sequences of *AeSWEET11a* and *AeSWEET11b* showed over 92% similarity (Table S2), strongly indicating that they are duplicate genes. However, they were located on different chromosomes (Figure 1) and failed to form a syntenic gene pair (Figure 4), pointing to their origination through alternative duplication mechanisms. The expression profiles of *AeSWEET11a* and *AeSWEET11b* were highly similar across seven distinct tissues and five fruit developmental stages, implying shared functionality under these conditions. Nevertheless, marked differences were observed in their intron lengths and promoter *cis*-elements (Figure 5). For example, *AeSWEET11a* contained the ARE and WUN-motif *cis*-elements, which respond to anaerobic induction and wound-responsive signals, respectively, whereas *AeSWEET11b* harbored TC-rich repeats and LTR elements, which mediate defense and stress responses and low-temperature responses, respectively. This divergence suggested potential functional specialization under varying stress conditions. Furthermore, oligomerization activates SWEET proteins, allowing for the formation of homomeric or heteromeric multimers to promote sugar transport [9]. In *Arabidopsis*, eight AtSWEET proteins (AtSWEET1, 5, 6, 8, 11, 12, 16, and 17) were capable of forming homomeric complexes, while 47 heteromeric combinations could arise among the 17 AtSWEET proteins [66]. Peach *PpSWEET11a* and *PpSWEET14*, both located in the plasma membrane and capable of sucrose transport, showed similar expression patterns in source and sink tissues [67]. Overexpressing either *PpSWEET11a* or *PpSWEET14* altered sucrose accumulation significantly, while co-expression led to an additive effect on sucrose levels, attributed to their ability to form homodimers and heterodimers, thereby enhancing transport activity [67]. Similarly, *AeSWEET11a* and *AeSWEET11b* were confirmed to have identical subcellular localization and sugar transport functions. Given their shared highly analogous expression patterns and strong correlation with sugar accumulation across fruit development and ripening stages, we hypothesized their synergistic potential to assemble into homodimers and heterodimers, collectively enhancing sugar transport efficiency throughout these processes. These findings suggested that *AeSWEET11a* and *AeSWEET11b* might serve as promising target genes or molecular markers for enhancing sugar accumulation in *A. eriantha* fruits through targeted breeding efforts.

## 4. Materials and Methods

### 4.1. Identification of AeSWEET Genes Through a Genome-Wide Analysis

The whole-genome sequence of *A*. *eriantha* Bentham ‘Midao 31’ was retrieved from the China National Center for Bioinformation (https://ngdc.cncb.ac.cn/gwh/Assembly/64038/show, accessed on 16 August 2024) [37]. The hidden Markov model (HMM) seed profile of SWEET domain (PF03083) was downloaded from the Pfam database (http://pfam.xfam.org/, accessed on 16 August 2024). Candidate *AeSWEET* genes were initially screened using HMMER v3.4 software (http://www.hmmer.org/, accessed on 16 August 2024), with an e-value threshold of <1 × 10^−5^. To comprehensively identify *AeSWEET* genes, protein sequences of AtSWEET were retrieved from the *Arabidopsis* Information Resource (TAIR, https://www.arabidopsis.org/, accessed on 16 August 2024). These AtSWEET sequences served as queries for homologous searches against all protein sequences of ‘Midao 31’ using the BLAST GUI Wrapper in TBtools-II v2.1 [68], yielding candidate genes. A Venn diagram was constructed to compare the results from HMMER and BLASTP, with the overlapping sequences identified as candidate *AeSWEET* genes. Subsequent validation using the Conserved Domains Database (https://www.ncbi.nlm.nih.gov/Structure/cdd/cdd.shtml, accessed on 16 August 2024) excluded sequences lacking or containing incomplete SWEET domains.

The analysis of AeSWEET protein properties was conducted using the ExPASy ProtParam tool (https://web.expasy.org/protparam/, accessed on 19 August 2024). Transmembrane topology prediction was performed using the Deep TMHMM-1.0 tool (https://services.healthtech.dtu.dk/services/DeepTMHMM-1.0/, accessed on 19 August 2024). Subcellular localization was assessed using ProtComp v.9.0 with default parameters (http://www.softberry.com, accessed on 19 August 2024).

### 4.2. Chromosomal Location and Phylogenetic Relationship of AeSWEETs

The chromosomal positions of *AeSWEET* genes were retrieved from the GFF file, and subsequently visualized using the Gene Location Visualize program in TBtools-II v2.1. The protein sequences of SWEET family in *V*. *vinifera* and *L*. *chinensis,* were obtained according to previous reports [41,42]. The interspecific phylogenetic tree was constructed utilizing SWEET protein sequences from *A*. *eriantha*, *A*. *thaliana*, *V*. *vinifera*, *L*. *chinensis* and *O. sativa*, while the intraspecific evolutionary relationships were resolved through phylogenetic analysis of 26 AeSWEET sequences. Multiple sequence alignments were performed using the Muscle algorithm, followed by precise trimming of non-conservative regions through Quick Run TrimAL. Using MEGA v12, maximum-likelihood phylogenetic trees were constructed with 1000 bootstrap replicates.

### 4.3. Gene Structure and Protein Domains of AeSWEETs

The exon-intron structure of *AeSWEET* genes was analyzed using annotated data retrieved from the GFF file. Conserved motifs and domains of AeSWEET proteins were identified, respectively, using the online tools of MEME (https://meme-suite.org/meme/, accessed on 26 August 2024) and CD-Search (https://www.ncbi.nlm.nih.gov/Structure/bwrpsb/bwrpsb.cgi, accessed on 26 August 2024). Finally, the visualization of gene-structures, conserved motifs, domains, and motif compositions was carried out using the Gene Structure View program and Batch MEME Motif Viz tool in TBtools-II v2.1.

### 4.4. Intraspecific and Interspecific Collinearity Analysis

Genome sequences of *S*. *lycopersicum*, *O*. *sativa*, and *S*. *bicolor* were downloaded from Ensembl Plants database (http://plants.ensembl.org/index.html, accessed on 28 August 2024), along with the *A*. *thaliana* genome from TAIR, to represent dicot and monocot plants in the collinearity analysis. Intraspecies and interspecies collinearity for 26 *AeSWEET* genes and *SWEET* genes from other plants were performed using the One-Step MCScanX tool integrated into TBtools-II v2.1. Based on the results of the intraspecies collinearity analysis, the Simple Ka/Ks Calculator was employed to compute Ka/Ks ratios for aligned duplicated gene pairs.

### 4.5. Cis-Acting Element Analysis of AeSWEET Promoters

The 2000 bp sequences upstream of the start codon of *AeSWEET* genes were extracted from the ‘Midao 31’ genome and defined as their promoter regions. These promoter sequences were subsequently analyzed for *cis*-acting elements using PlantCARE (http://bioinformatics.psb.ugent.be/webtools/plantcare/html/, accessed on 12 September 2024). Finally, the categories and quantities of identified *cis*-acting elements were categorized and quantified using the Excel and visualized with GraphPad Prism 9.0.

### 4.6. Plant Materials and Quantitative Real-Time PCR

The five-year-old *A*. *eriantha* Bentham ‘Lvyan 3’ vines cultivated at the experimental base of the Fujian Academy of Agricultural Sciences (26°23′ N, 117°9′ E) in Mingxi County, Fujian Province, China, served as experimental materials. To assess gene expression across various tissues, mature leaves and annual stems were collected on 20 April 2024, while flowers were sampled on 8 May 2024. Fruit tissues (epicarp, outer pericarp, inner pericarp, and core) were collected on 20 October 2024, from fruits that had reached harvest maturity (with a total soluble solids content of 8.0 ± 0.5% and a dry matter content of 19.6 ± 0.8%). For the analysis of gene expression throughout fruit development and ripening, fruits were collected at five developmental stages: 60 days after full bloom (DAF), 90 DAF, 135 DAF, 165 DAF (harvest maturity), and 15 days after harvest (DAH; reaching the edible stage with a total soluble solids content of 18.5 ± 0.9% and a firmness of 1.2 ± 0.3 N). Fruit maturity was assessed based on the total soluble solids, dry matter content, and firmness. Total soluble solids were measured using a digital hand-held refractometer (Atago, Tokyo, Japan). Dry matter content was determined by drying 3 mm thick equatorial fruit slices at 60 °C to a constant weight (approximately 24 h). Fruit firmness was assessed with a TMS-Pilot texture analyzer (FTC, Sterling, VA, USA). Nine vines were divided into three groups serving as three biological replicates. Each biological replicate contained twelve fruits of uniform size and free from damage.

The RNAprep Pure Plant Plus Kit (Tiangen, Beijing, China) was employed to extract total RNA of kiwifruit tissues. RNA integrity and concentration were analyzed using 1% agarose gels and a NanoDrop One Spectrophotometer (Thermo Scientific, Waltham, MA, USA). 500 ng of total RNA per sample was subsequently reverse-transcribed into complementary DNA (cDNA) for quantitative real-time PCR (qRT-PCR) using the HiScript III RT SuperMix for qPCR (gDNA wiper plus; Vazyme, Nanjing, China). Each *AeSWEET* gene primer was designed with the assistance of online PrimerQuest Tool (https://sg.idtdna.com/PrimerQuest/Home/Index, accessed on 30 October 2024), and their specificity was verified through the primer check function in TBtools-II v2.1. qRT-PCR was carried out using a LightCycler480 Real-time instrument (Roche, Mannheim, Schaffhausen, Switzerland) and the Taq Pro Universal SYBR qPCR Master Mix (Vazyme, Nanjing, China), in accordance with the manufacturer’s instructions. Using five 10-fold dilutions of the cDNA mixture from the test samples as templates, qRT-PCR amplification was performed for each pair of primers, and standard curves were generated to analyze their amplification efficiencies. The *AeActin* gene (Aermda05g007346) of *A*. *eriantha* served as a housekeeping gene, and relative expression levels of *AeSWEET* genes were calculated using the Pfaffl analysis method [69]. Each sample was analyzed with three biological replicates and three technical replicates. Detailed information regarding *AeSWEET* primers was provided in Table S4.

### 4.7. Measurement of Sucrose, Glucose and Fructose

Sucrose, glucose, and fructose concentrations were quantified using high-performance liquid chromatography (HPLC) with methodological modifications [62]. Briefly, fruit samples were homogenized in liquid nitrogen, and 2 g of the resulting powder was then transferred to a 50 mL centrifuge tube. After adding 15 mL of 80% (*v*/*v*) ethanol and mixing thoroughly, the mixture was extracted by ultrasonication at 35 °C for 30 min. After cooling to room temperature, the mixture was centrifuged at 4000 rpm for 10 min, and the supernatant was transferred to a volumetric flask. The residue was re-extracted once, and the supernatants were combined. The combined supernatants were subsequently dried by rotary evaporation, and an equal volume of deionized water was then added to dissolve the dried extract. The fully dissolved solution was filtered through a 0.22 µm membrane, and the resulting filtrate was used for sugar analysis via HPLC. Chromatographic separations were performed on an Agilent ZORBAX NH2 column (4.6 mm × 250 mm, 5.0 µm), with a mobile phase consisting of acetonitrile and deionized water (70:30, *v*/*v*). The injection volume was 20 µL, the flow rate was 1.0 mL/min, and the column temperature was maintained at 40 °C. Detection was performed using a refractive index detector (RID). Each sample was analyzed with three biological replicates.

### 4.8. Subcellular Localization

The open reading frame (ORF) sequences of *AeSWEET11a* and *AeSWEET11b* were, respectively, amplified from fruits of ‘Lvyan 3’ at 165 DAF using specific primers (AeSWEET11a-OrfF/R and AeSWEET11b-OrfF/R; Table S5), and subsequently cloned into the pEASY^®^-Blunt zero vector (TransGen, Beijing, China). Six clones for each gene were selected via PCR and confirmed through sequencing. The validated vectors were then employed as templates to amplify the coding sequences of *AeSWEET11a* and *AeSWEET11b* without the stop codons under the primers AeSWEET11a-SubF/R and AeSWEET11b-SubF/R (Table S5), respectively. The purified PCR products were fused to the N-terminus of GFP within 62SK vector, utilizing the ClonExpress Ultra One Step Cloning Kit (Vazyme, Nanjing, China) to generate constructs of 35S:AeSWEET11a-GFP and 35S:AeSWEET11b-GFP. The empty 62SK vector (35S:GFP) was employed as a control. Additionally, the 35S:AtNAA60-mKate vector served as a plasma membrane marker [38,70].

The vectors 35S:AeSWEET11a-GFP and 35S:AtNAA60-mKate, 35S:AeSWEET11b-GFP and 35S:AtNAA60-mKate, as well as 35S:GFP and 35S:AtNAA60-mKate, were co-transformed into *N*. *benthamiana* leaves via *Agrobacterium*-mediated infiltration according to reported methods [66]. Following infiltration, the tobacco plants were incubated for an additional 3 days. Fluorescence imaging was then performed using a Nikon C2-ER confocal microscope. GFP, mKate, and chloroplast autofluorescence were excited at 488 nm, 561 nm, and 640 nm, with emission detected at 510 nm, 580 nm, and 675 nm, respectively.

### 4.9. Yeast Mutant Complementary Growth Assay

The coding sequences of *AeSWEET11a* and *AeSWEET11b* were amplified using specific primers, AeSWEET11a-ExpF/R and AeSWEET11b-ExpF/R (Table S6), respectively, and subsequently inserted into the pDR195 vector between BamHI and XhoI sites via homologous recombination using the ClonExpress Ultra One Step Cloning Kit (Vazyme, Nanjing, China). The recombinant constructs were then cloned into DH5a cells. Six colonies were selected based on PCR screening and confirmed by sequencing.

To transform yeast, 1 µg of the verified recombinant plasmid was combined with 100 μL of competent yeast cells and 500 μL of PEG/LiAc solution. Following a 30 min incubation at 30 °C in a water bath, 20 μL DMSO was added, and the mixture was heat-shocked at 42 °C water bath for 20 min. The transformation mixture was centrifuged at 700 g to remove the supernatant, and 1 mL of YPM liquid medium was added before shaking at 30 °C for 90 min. After another centrifugation at 700 g for 5 min, the yeast was resuspended in 1ml 0.9% (*w*/*v*) NaCl. The EBY.VW4000 yeast strain expressing *AeSWEET11a* or *AeSWEET11b* was cultivated on SD/-Ura medium supplemented with 2% (*w*/*v*) maltose, while the SUSY7/ura3 strain was grown on SD/-Ura medium containing 2% (*w*/*v*) glucose. After 3-day incubation at 30 °C, individual colonies were verified by PCR. The confirmed strains were resuspended in 0.9% NaCl, and when the optical density at 600 nm (OD_600_) reached 0.8, cultures were diluted to 10^0^, 10^−1^, 10^−2^, and 10^−3^ and subsequently spotted onto the SD/-Ura medium supplemented with various sugar sources, including 2% (*w*/*v*) maltose, fructose, glucose, galactose and sucrose. Following incubation at 30 °C for 88 h, the strains’ growth was evaluated. The strain harboring SlSWEET14-pDR195 was used as positive control [65]. For each group, three sequencing-verified clones were selected to serve as three biological replicates. Each was independently transformed into yeast and spotted onto SD/-Ura medium supplemented with various sugar sources.

### 4.10. Statistical Analyses

Data normality was tested using GraphPad Prism 9.0 (Normality and Lognormality Tests). Differences in *AeSWEET* gene expression levels and sugar concentrations were analyzed by one-way ANOVA with Tukey’s Honestly Significant Difference test. Correlations between transcriptional levels of *AeSWEET* genes and sugar contents (sucrose, fructose, glucose) were determined by calculating Pearson correlation coefficients using the software’s analytical suite, with a two-tailed significance test employed to verify statistical relevance.

## Figures and Tables

**Table 1:** Basic physical and chemical characteristics of AeSWEET proteins.

Protein Name	Amino Acids (aa)	Molecular Weight (Da)	Isoelectric Point	Instability Index	GRAVY	TMH	Subcellular Localization
AeSWEET1a	251	27,425.63	9.35	33.64	0.596	7	PM
AeSWEET1b	252	27,780.02	9.47	33.1	0.508	7	PM
AeSWEET1c	252	27,485.73	9.68	28.76	0.636	7	PM
AeSWEET2a	236	25,895.9	9.24	38.06	0.883	7	PM
AeSWEET2b	236	26,221.08	8.94	41	0.783	7	PM
AeSWEET3a	212	23,971.52	9.7	38.24	0.250	7	PM
AeSWEET3b	243	27,228.28	9.73	24.97	0.519	7	PM
AeSWEET4a	198	21,746.05	9.72	33.39	0.703	7	PM
AeSWEET4b	238	26,245.35	9.52	34.27	0.713	7	PM
AeSWEET5	234	26,451.54	8.66	36.72	0.65	7	PM
AeSWEET6a	305	34,066.5	9.62	39.75	0.518	7	PM
AeSWEET6b	219	24,547.16	8.92	42.66	0.616	6	PM
AeSWEET6c	279	31,541.67	9.48	36.55	0.569	7	PM
AeSWEET7	259	28,591.22	9.71	45.36	0.59	7	PM
AeSWEET10a	287	32,095.65	9.39	39.72	0.802	7	PM
AeSWEET10b	290	32,615.36	8.15	39.24	0.85	7	PM
AeSWEET10c	290	32,593.16	9.03	39.01	0.751	7	PM
AeSWEET11a	275	30,940.75	8.47	37.23	0.627	7	PM
AeSWEET11b	275	30,917.98	8.37	38.51	0.719	7	PM
AeSWEET12	281	31,991.57	9.25	40.11	0.29	7	PM
AeSWEET15	287	32,096.05	7.04	36.89	0.671	7	PM
AeSWEET16a	249	27,717.86	9.26	40.34	0.417	5	PM
AeSWEET16b	304	33,148.24	9.65	33.44	0.377	7	PM
AeSWEET17a	240	26,569.19	6.5	48.66	0.588	7	PM
AeSWEET17b	253	27,794.08	9.15	36.01	0.734	7	PM
AeSWEET17c	244	26,962.85	7.77	35.7	0.674	7	PM

Note: GRAVY = the grand average of hydropathy. TMH = transmembrane helix number. PM = plasma membrane.

## Data Availability

All datasets generated and analyzed during this study are available from the corresponding author on reasonable request.

## Supplementary Materials

The following supporting information can be downloaded at: https://www.mdpi.com/article/10.3390/plants14203140/s1, Figure S1: Compositions of the 14 motifs identified in the AeSWEET family; Table S1: Gene information of the *AeSWEET* family; Table S2: Pairwise similarities among *AeSWEET* family genes; Table S3: Ka/Ks ratios of homologous genes within the *AeSWEET* family; Table S4: Primer sequences used for qRT-PCR; Table S5: Primer sequences used for subcellular localization; Table S6: Primer sequences used for functional analysis.
