# Supplementary figures and images for: Comprehensive Identification and Expression Analysis of the SWEET Gene Family in Actinidia eriantha Reveals That Two AeSWEET11 Genes Function in Sucrose and Hexose Transport
Source: Plants (Basel). 2025 Oct 11;14(20):3140. doi: 10.3390/plants14203140 (PMC12567542; doi:10.3390/plants14203140)

## Supplementary Figures

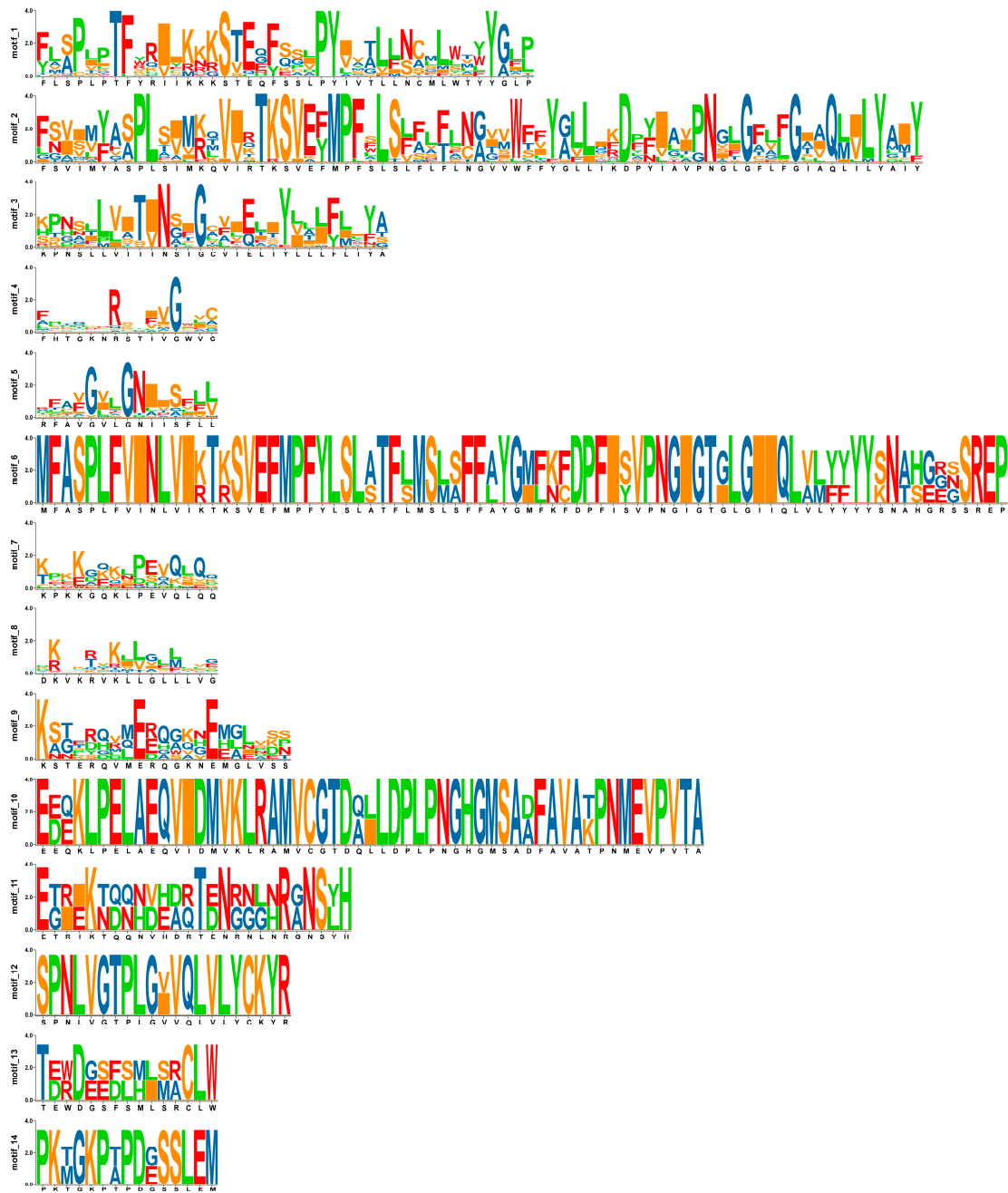

**Figure S1.** Compositions of the 14 motifs identified in the AeSWEET family.

Supplement: Supplementary file 1 [file plants-14-03140-s001.zip › Supplementary_Figures.pdf]
